# Supplementary material for: Enhanced fetal hematopoiesis in response to symptomatic SARS-CoV-2 infection during pregnancy
Source: Commun Med (Lond). 2023 Dec 11;3:177. doi: 10.1038/s43856-023-00406-6 (PMC10713620; doi:10.1038/s43856-023-00406-6)
Supplement: Supplementary file 1 — Supplementary Information [file 43856_2023_406_MOESM1_ESM.pdf]

Supplementary Figures legends

Supplementary Figure 1: Influence of the offspring's gender and of COVID-19 on cord blood mononuclear cells transcriptome.

a. PCA plot of all samples based on the 500 most expressed genes. Data are color-coded and shape-coded with the offspring's gender and COVID-19 status.

b. Hierarchical clustering analyses performed using DESeq2 between COVID+S and COVID-/+A samples. Color code (from dark blue to green) refers to the distance metric used for clustering (dark blue corresponds to the maximum of correlation values).

c. Volcano plot showing DEG in COVID+A samples compared with COVID- samples.

Data information: In (a-c), RNA-seq was performed in cord blood mononuclear cells from COVID- (n=5 patients), COVID+A (n=3 patients) and COVID+S mothers (n=9 patients) harvested at delivery. An adjusted p-value below 0.05 was considered statistically significant.

Supplementary Figure 2: Extended flow cytometry analyses of cord blood mononuclear cells in uninfected and symptomatic COVID-19 patients.

a. Gating strategy for the identification of the different hematopoietic progenitors: hematopoietic stem and progenitor cells (HSPC), multipotent progenitor cells (MPP), lymphoid-primed multipotent progenitor (LMPP), multi-lymphoid progenitor (MLP), granulocyte-monocyte progenitors (GMP), common myeloid progenitors (CMP), megakaryocyte-erythrocyte progenitors (MEP) and B/NK progenitors (BNKPro).

b-e. Quantification of CMP (b), LMPP (c), MLP (d) and BNKPro (e) subpopulations as percentages of CD34+ cells in COVID- (n=3 patients) and severe COVID+S (n=3 patients) cord blood mononuclear cells. Data are presented as means with SEM and individual values. Statistical analyses were performed with two-tailed unpaired t-test (b, d-e) or two-tailed

unpaired t-test with Welch's correction (c). (ns) not significant. Source data are available in Supplementary Data 1.

Supplementary Figure 3: A signature of erythropoiesis with features of hypoxic signaling is observed in COVID+S samples.

Heatmap displaying the expression of the defining genes of the 10 modules defined in[34] (Supplementary Data 3) between COVID+S and COVID-/+A samples. Mean fold change (FC) in COVID+S versus COVID-/+A samples, and adjusted p-values (Padj) are shown.

Supplementary Figure 4: Uncropped versions of the western blots.

a-b. Uncropped versions of the western-blot of VHL (a) and ACTIN (b) presented in Fig. 5c.

Supplementary Tables legends

Supplementary Table 1: RNA Integrity Numbers and sequencing depths (in millions of reads) of RNAseq samples.

Supplementary Table 2: List of antibodies used.

Supplementary Data legends

Supplementary Data 1: Source data of Figures 2b-f, 3c-e, 4a-i, 5a-c, and Supplementary Figure 2b-e.

Supplementary Data 2: Defining genes of the 9 modules displayed in Zheng, S. et al. Mol. Syst. Biol. 2018 (reference 26).

Supplementary Data 3: Defining genes of the 10 modules displayed in Bernardes, J. P. et al. Immunity 2020 (reference 34).

Supplementary Figure 1

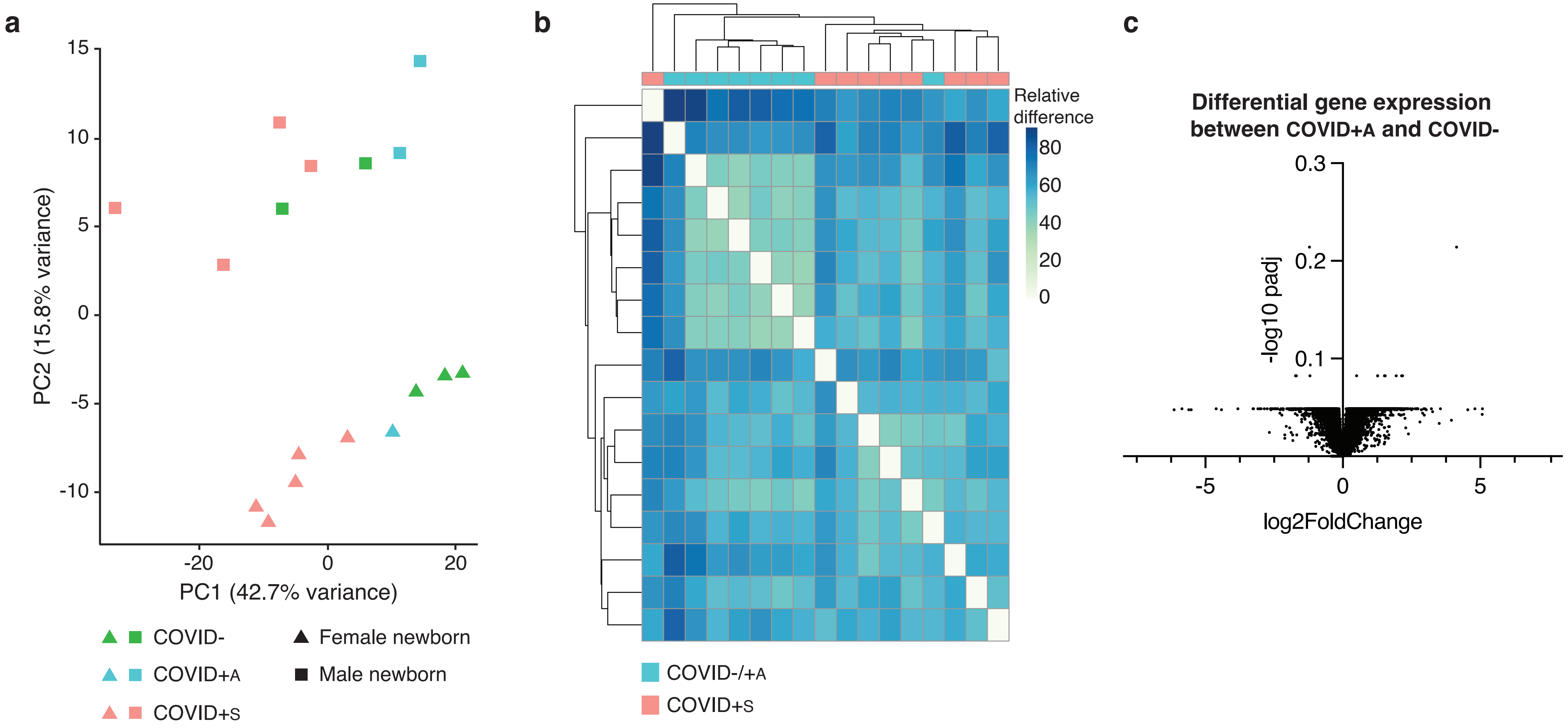

Supplementary Figure 2

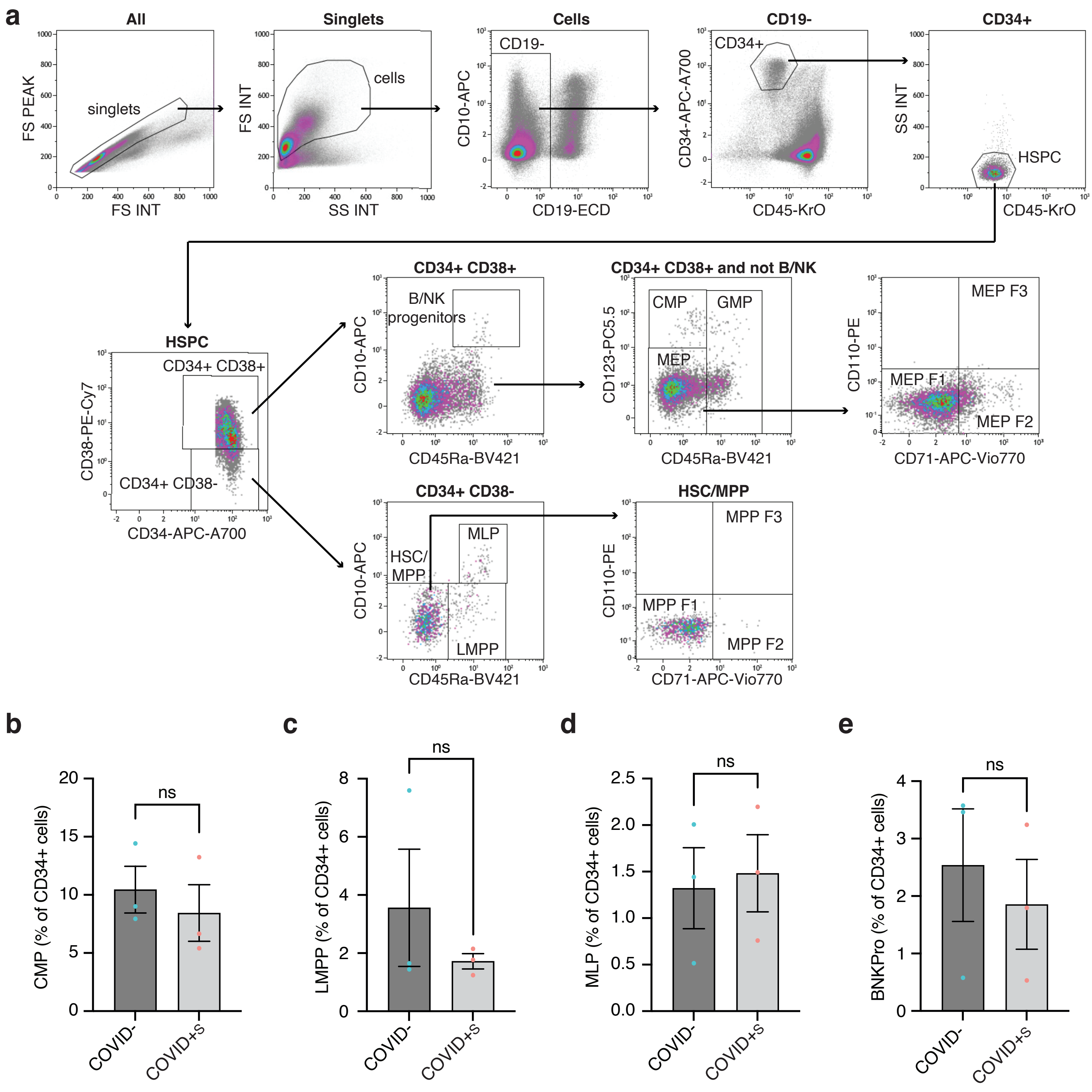

Supplementary Figure 3

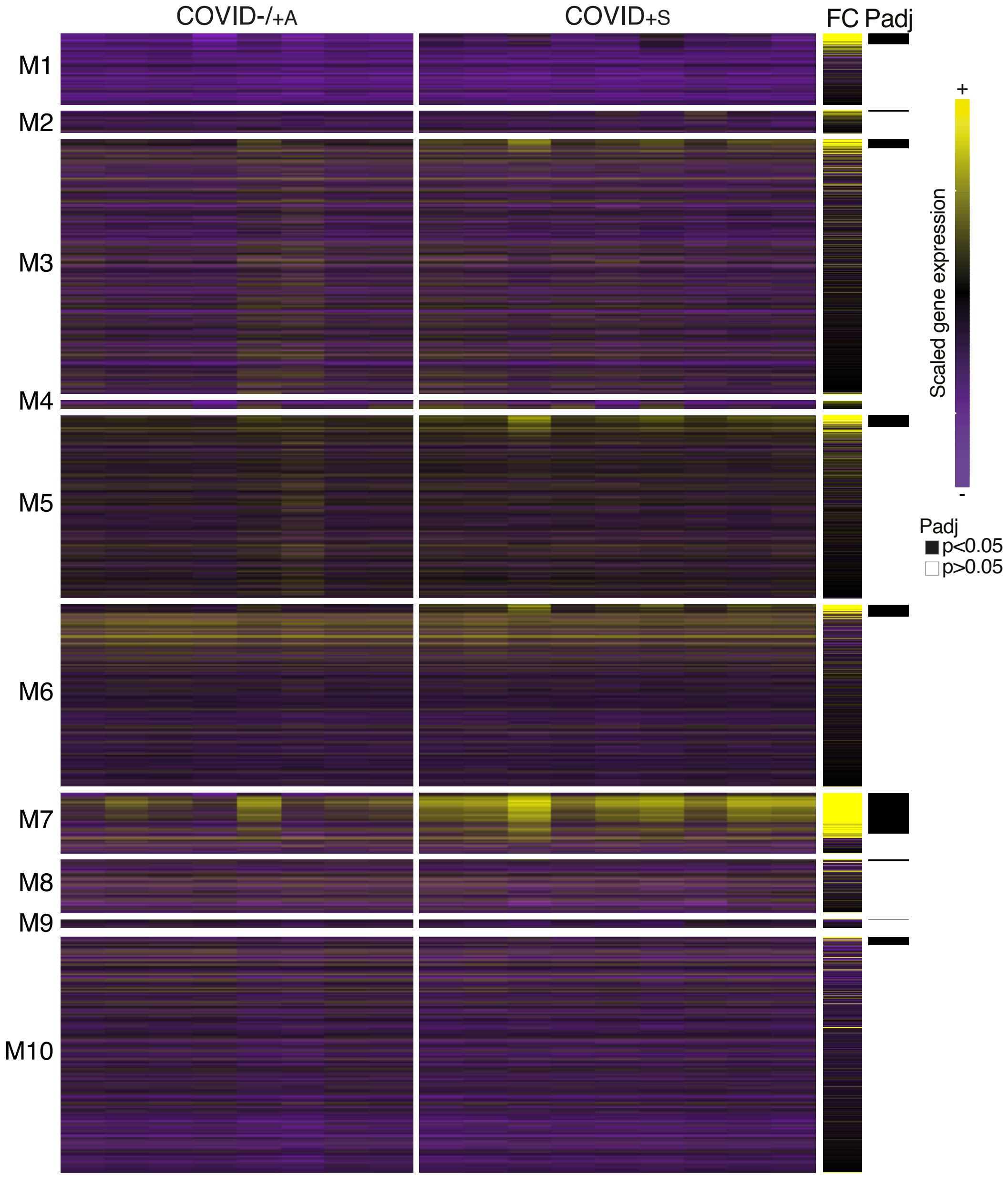

Supplementary Figure 4

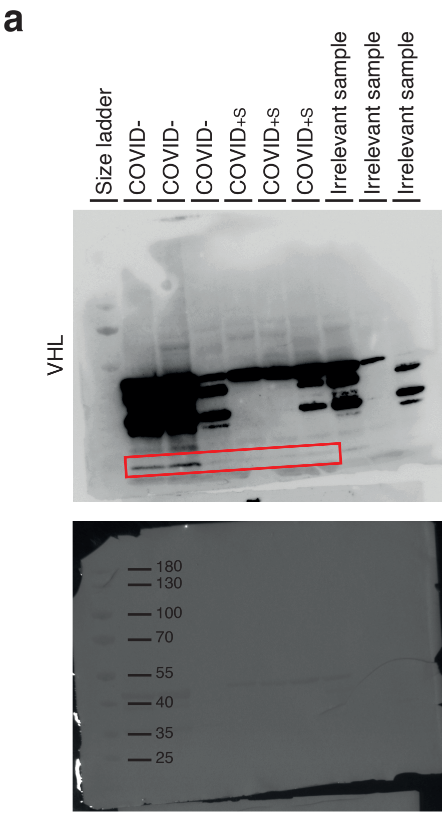

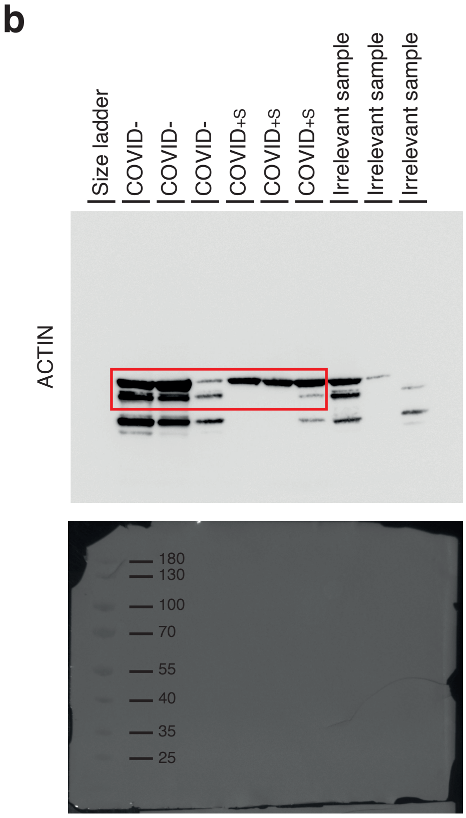

Supplementary Table 1: **RNA Integrity Numbers and sequencing depths (in millions of reads) of RNAseq samples.**

Sample	RNA Integrity Number	Sequencing depth (millions of reads)
COVID- 1	9.4	48.3
COVID- 2	9.6	42.7
COVID- 3	9.6	46.5
COVID- 4	9.2	43.3
COVID- 5	9.9	30.8
COVID+A 1	7.7	28.0
COVID+A 2	9.5	43.8
COVID+A 3	9.8	50.0
COVID+S 1	9.6	33.9
COVID+S 2	9.8	33.0
COVID+S 3	9.4	46.2
COVID+S 4	9.5	40.0
COVID+S 5	9.5	50.3
COVID+S 6	9.7	43.8
COVID+S 7	9.1	29.6
COVID+S 8	10.0	28.6
COVID+S 9	9.9	25.0

Supplementary Table 2: List of antibodies used.

Antibody	Reference	Clone
CD110-PE	562159 (Becton Dickinson)	1.6.1
CD36-FITC	B49201 (Beckman Coulter)	FA6.152
CD19-ECD	A07770 (Beckman Coulter)	J3-119
CD123-PC5.5	B20022 (Beckman Coulter)	SSDCLY107D2
CD38-PE-Cy7	335825 (Becton Dickinson)	HB7
CD10-APC	130-114-503 (Miltenyi Biotec)	REA877
CD34-APC-A700	B92417 (Beckman Coulter)	581
CD71-APC-Vio770	130-126-306 (Miltenyi Biotec)	AC102
CD45Ra-BV421	562885 (Becton Dickinson)	HI100
CD45-KrO	B36294 (Beckman Coulter)	J33
