# Supplementary material for: Enhanced fetal hematopoiesis in response to symptomatic SARS-CoV-2 infection during pregnancy
Source: Commun Med (Lond). 2023 Dec 11;3:177. doi: 10.1038/s43856-023-00406-6 (PMC10713620; doi:10.1038/s43856-023-00406-6)
Supplement: Supplementary file 2 — Description of Additional Supplementary Files [file 43856_2023_406_MOESM2_ESM.pdf]

Description of Additional Supplementary Files

File Name: Supplementary Data 1

Description: Source data of Figures 2b-f, 3c-e, 4a-i, 5a-c, and Supplementary Figure 2b-e.

File Name: Supplementary Data 2

Description: Defining genes of the 9 modules displayed in Zheng, S. et al. Mol. Syst. Biol. 2018 (reference 26).

File Name: Supplementary Data 3

Description: Defining genes of the 10 modules displayed in Bernardes, J. P. et al. Immunity 2020 (reference 34).
